# Selenium Biofortification and an *Ecklonia maxima*-Based Seaweed Extract Jointly Compose Curly Endive Drought Stress Tolerance in a Soilless System

**DOI:** 10.3390/plants15010170

**Published:** 2026-01-05

**Authors:** Beppe Benedetto Consentino, Fabiana Mancuso, Lorena Vultaggio, Pietro Bellitto, Georgia Ntatsi, Claudio Cannata, Gaetano Giuseppe La Placa, Rosario Paolo Mauro, Salvatore La Bella, Leo Sabatino

**Affiliations:** 1 Department of Agricultural, Food and Forestry Sciences, University of Palermo, Viale delle Scienze Building 4, 90128 Palermo, Italy; beppebenedetto.consentino@unipa.it (B.B.C.); fabiana.mancuso@unipa.it (F.M.); lorena.vultaggio@unipa.it (L.V.); pietro.bellitto@unipa.it (P.B.); gaetanogiuseppe.laplaca@unipa.it (G.G.L.P.); salvatore.labella@unipa.it (S.L.B.); 2 Department of Crop Science, Laboratory of Vegetable Production, Agricultural University of Athens, 11855 Athens, Greece; ntatsi@aua.gr; 3 Department of Agriculture, Food and Environment (Di3A), University of Catania, Via Valdisavoia, 5, 95123 Catania, Italy; claudio.cannata@unict.it (C.C.); rosario.mauro@unict.it (R.P.M.)

**Keywords:** relative water content, cell membrane stability marker, stress alleviators, protected environment, biostimulant, water shortage, *Cichorium endivia*

## Abstract

Vegetable cultivation is currently facing complex challenges related to climate change, with negative repercussions on plant performance. In this scenario, the employment of eco-friendly agronomic tools capable of boosting plant tolerance to abiotic stresses is fundamental. Among them, the use of non-microbial biostimulants, such as seaweed extracts (SwEs), and microelements, like selenium (Se), is considered an efficient approach to overcome abiotic stresses. In this experiment, the performance of chicory plants cultivated under three different irrigation levels (100%, 75% or 50% of substrate water holding capacity) and treated with SwE, Se or their combination (SwE + Se) was evaluated. The results revealed that drought stress significantly decreased growth, productivity and relative water content but increased soluble solid content, dry matter percentage, and proline and malondialdehyde concentrations. The application of Swe, Se or Swe + Se enhanced growth, productive features and soluble solid content and reduced dry matter percentage, proline and malondialdehyde compared to the control. Based on our results, Se and SwE combined application could be a valuable approach to face moderate drought stress on curly endive plants and improve productive and quality traits.

## 1. Introduction

Vegetable crops represent around 12% of the global yearly agricultural production (9 billion tons) [1]. Considering the nutritional and functional role of vegetables, their consumption has significantly increased over the past few years [2]. Leafy vegetables are an important font of fiber, vitamins, nutrients and phytochemicals that positively affects human health [3]. *Cichorium endivia* L., belonging to the *Asteraceae* family, is one of the most consumed salads worldwide appreciated for its content of numerous beneficial metabolites, such as unsaturated fatty acids, alkaloids, coumarins, flavonoids, and tannins [4,5]. Although mild drought stress could have a helpful effect on leafy vegetables by improving secondary metabolite production (e.g., polyphenol, flavonoids and ascorbic acid) [6], severe and prolonged water deficiency conditions lead to significant reductions in vegetable production and quality [7]. In this regard, changes in climate conditions have caused a progressive temperature increase and a reduction in rainfall [8]. Drought is widely recognized as one of the major abiotic stresses that has a harmful effect on plant development and crop production. It is predicted that by the end of the 21st century, arid and semi-arid regions could represent over 50% of the Earth’s surface [9]. As the frequency of adverse weather conditions increases, research efforts focusing on crop tolerance to abiotic stress, with reference to drought tolerance, should increase. Crop responses to abiotic stress conditions are closely linked to plant species and to the severity and duration of the distress, which in turn can cause several alterations in plant physiology and metabolism [10]. Water deficits alter numerous morphological and physiological mechanisms such as chlorophyll content, stomatal closure mediated by abscisic acid (ABA), leaf senescence, cell expansion, leaf area, nutrient uptake, photosynthesis rate, metabolic balance and stomatal conductance [11,12,13]. Moreover, insufficient water availability causes osmotic and oxidative stress in vegetables, inducing reactive oxygen species (ROS) overproduction and accumulation that leads to cell damage, protein structure destruction and malondialdehyde (MDA) accumulation [14,15]. In abiotic stress conditions, vegetable crops trigger multifaceted mechanisms, such as the regulation of several key gene signaling pathways, the elicitation of antioxidant system activity, and an increase in protein synthesis [16,17]. However, these mechanisms are not enough to tolerate a long drought period [15]. In the present scenario, abiotic stress represents a growing threat to the sustainability of vegetable production, requiring innovative strategies to improve horticultural sector resilience. According to the EU Commission guidelines, biostimulants are considered a sustainable tool capable of mitigating abiotic stress and improving crop production and the quality standards of vegetables [18,19]. Biostimulants are classified as substances able to promote plant growth and resource use efficiency and enhance abiotic stress tolerance and crop quality features via direct and indirect mechanisms that affect plant metabolism [20,21]. Among non-microbial biostimulants, seaweed extracts (SwEs) represent a class of promising products used to overcome drought stress [22] due to their heterogeneous content of bioactive substances such as nutrients, amino acids, vitamins, hormones, betaines, polyamines and alginates that act synergically [23,24]. Recent studies have shown that SwE treatment via foliar spray or fertigation positively influences the relative water content (RWC), water use efficiency (WUE), stomatal conductance and transpiration rate of plants grown under drought stress conditions [25]. As reported by Campobenedetto et al. [24], tomato plants grown under water deficiency conditions and supplied with SwE showed a lower content of abscisic acid, malondialdehyde and proline linked to lower ROS scavenger enzyme activity compared to non-biostimulated tomato. These results evidenced a significant reduction in oxidative stress level. Recent studies [26] have associated the potential mode of action of SwE with the regulation of some genes implicated in the plant stress response, such as those related to pigment biosynthesis and the antioxidant system. On the other hand, selenium (Se) is considered a crucial micronutrient in human nourishment, even though it is not considered indispensable to plants [27,28]. Several studies report a defensive role of selenium against redox imbalance by regulating numerous processes to improve plant tolerance and preserve cells and tissue [29]. Moreover, Se acts by modulating antioxidant enzyme (e.g., glutathione peroxidase) activity and decreasing lipid peroxidation in plants [30]. Additionally, Se safeguards chloroplasts and increases chlorophyll amount under low water availability [31]. According to Rady et al. [15], who investigated the effects of Se application on modulating drought stress in tomato, Se application induced improvements in plant growth traits, highlighting its crucial role in the regulation of metabolic and enzymatic processes. This result could be linked to the increase in starch accumulation in the chloroplasts and to the stimulation of water uptake through better root activity under stress conditions [32]. Other studies have reported the positive role of this microelement in stimulating growth traits in soybean, eggplant and common buckwheat [33,34,35]. Comparable results are reported by Zhong et al. [36] who also investigate the effects of Se supply on tomato grown under drought conditions. The authors underlined that the application of Se (2.5 mg L^−1^) caused a reduction in MDA concentration (−21.5%), an increase in the net photosynthetic rate (+111.8%), and an upsurge in dry matter content (+29.0%) compared to non-treated plants. Since the application of Se or SwE can exert a positive effect on plant tolerance to drought stress, our hypothesis is that their combination could be more effective (producing an additive or synergistic effect) than their single application for enhancing plant performance in stressful conditions. Consequently, the present research assessed the effects of the foliar spray application of Se, SwE and their combination (SwE + Se) on ‘Wallaonne’ *Cichorium endivia* cultivated under different water levels (50, 75 and 100% of substrate water holding capacity), studying plant quantitative and qualitative responses.

## 2. Materials and Methods

### 2.1. The Field Experiment and Plant Material

During the 2024 winter season, a greenhouse experiment was conducted at the Department of Agriculture Food and Forestry Science (SAAF) of the University of Palermo (38°06′28″ N, 13°21′03″ E, 49 m a.s.l.). This study was accomplished in a pot soilless system, and we performed the experiment by using standardized growing media (a standard substrate mixture rather than soil). The commercial variety of curly endive ‘Wallone’ was transplanted on 8 February 2024 in plastic pots (Ø 21 cm) containing a peat/coconut fiber mix (40/60, *v*/*v*) and arranged to reach a final density of 10 plants m^−2^. To guarantee the nutritional requirements of the crop, a combination of Nitrosol 34 (34-0-0), monopotassium phosphate MKP (0-52-34) and Mugasol Mix (mixture of B, Cu, Fe, Mn and Zn, Mugavero Fertilizers^®,^, Termini Imerese, Italy) was applied weekly to achieve a final rate of 120 kg ha^−1^, 120 kg ha^−1^ and 200 kg ha^−1^, respectively. The application was made via fertigation at three different moments of the crop cycle: 10 and 21 days after transplant and 1 week before harvest. Maximum and minimum temperature and relative humidity were recorded using a data logger and the data are reported in Figure S1.

### 2.2. Experimental Design and Statistical Analysis

The experimental design was a randomized complete block design with 12 treatments and 3 replicates of 10 plants each, giving a total of 360 plants. Each treatment was randomized assigned within each of the 3 blocks to minimize the effect of spatial variability inside the greenhouse. Each treatment was characterized by the combination of three irrigation management (I) and drought alleviator treatments (T). The levels for I were defined as well watered (ww; 100% WHC), moderate drought stress (mds; 75% WHC) and severe drought stress (sds; 50% WHC), whereas the levels for T were control, SwE, Se and Swe + Se. Control treatments without biostimulants or biofortification were included to assess the specific effects of SwE or Se supplementation on plant growth and quality features.

A combined test of variance (ANOVA) was used to evaluate the impact of the irrigation management and drought alleviators on yield and quality traits. When significant, differences among mean values of the treatments were compared (*p* value ≤ 0.05). The analysis was performed by using SPSS software package version 14.0 (StatSoft, Inc., Chicago, IL, USA).

### 2.3. Substrate Water Retention Curve and Water Levels

A water retention curve of the substrate was obtained, according to the method of Markoska et al. [37], to characterize the hydraulic properties. Subsequently, the data were interpolated using the Van Genuchten model [38] to obtain the relation between the volumetric water content and the matrix potential. The volumetric water content was monitored daily using dielectric sensors (WET 150 Meter—Delta-T Devices, Cambridge, UK), specifically calibrated for the substrate used. Sensors were positioned at 5–7 cm depth in the central part of the pot, near the root system. For the first 10 days after transplant, plants were kept at 100% of the substrate WHC. After that, plants were grown under three different irrigation management strategies, ensuring 100%, 75% or 50% of the substrate WHC. To maintain the water supply, dielectric sensors were used, and the required amount of water to maintain the established WHC was supplied.

### 2.4. Biostimulant and Selenium Treatments

A non-microbial biostimulant was applied using a commercial seaweed extract (SwE) of *Ecklonia maxima* (Kelpstar^®^, Mugavero Fertilizzanti, Palermo, Italy). The SwE employed was manufactured through a cold micronization process to avoid material property alterations. The product was composed of 1% of organic nitrogen, 10% of organic carbon, phytohormones (11 mg L^−1^ of auxins and 0.03 mg L^−1^ of cytokinins), and 30% of organic compounds with a nominal molecular weight < 50 kDa. The recommended dose of 3 mL L^−1^ was administered for each application. Selenium (Se) was administered as sodium selenate (Na_2_SeO_4_) (Sigma-Aldrich, Saint Louis, MO, USA) at a dose of 8.0 µmol L^−1^. All treatments were supplied every 10 days, starting 1 week before transplant. The application of treatments (SwE, Se or SwE + Se) was accomplished via foliar spray, using 0.5 L m^−2^ of solution. Plants not treated with the biostimulant or with Se (sprayed with only water) served as controls.

### 2.5. Morphological, Physiological and Soluble Solid Content Measurements

The harvest took place on 3 April 2024 after the transplanting date at the commercial maturity of the crop. Immediately after the harvest, yield, plant height, root collar diameter, and leaf number were recorded for all plants. For dry matter determination and soluble solid content (SSC) analysis, five plants, randomly selected from each replicate, were employed. Head dry matter content was determined by dehydrating the samples in a forced-air oven (Memmert, Standard Series, Venice, Italy) at 80 °C for 24 h, followed by drying at 105 °C until a constant weight was reached. For the determination of SSC, leaf tissues were pressed to extract juice, which was then filtered. Subsequently, the SSC was determined using a digital refractometer (HI96801, Hanna Instruments, Padova, Italy).

### 2.6. Mineral Composition of Curly Endive

Total nitrogen (N) content in curly endive tissues was assessed using the Kjeldahl method.

Calcium (Ca), magnesium (Mg) and potassium (K) contents were determined by using atomic absorption spectroscopy (SavantAA, 200 ERRECI, Milan, Italy) [39], whereas phosphorus content was determined colorimetrically.

Regarding sulfur (S) content, 500 mg of dried tissue was digested with 4.0 mL of concentrated nitric acid (HNO_3_) and 2.0 mL of perchloric acid (HClO_4_) (Sigma–Aldrich, Saint Louis, MO, USA) at 120 °C for 1 h, followed by heating at 220 °C until perchloric acid fumes were observed. Total sulfur content was then measured using an atomic absorption spectrophotometer (SavantAA, 200 ERRECI, Milan, Italy).

Se concentration was determined by digesting 25 mg of dried leaf tissue with 2.5 mL of concentrated HNO_3_ and 1 mL of H_2_O_2_ in a microwave digestion system. The resulting solution was diluted to 25 mL with deionized water, and selenium content was measured using inductively coupled plasma mass spectrometry (ICP-MS) (Plasma Quant MS Elite, Jena, Germany), following the method described by Pedrero et al. [40].

### 2.7. Leaf Relative Water Content and Malondialdehyde and Proline Concentrations

At the end of the experiment, the relative water content (RWC) was evaluated through the procedure described by Toscano et al. [41], using the formula

$$\mathrm{RWC}=\left(\frac{lfm-ldm}{ltm-ldm}\right)\times 100$$

where lfm is the leaf disk fresh mass, ldm is the leaf disk dry mass and ltm is the leaf disk turgid mass.

Malondialdehyde (nmol g^−1^ fw) determination was accomplished following the Li et al. [42] protocol, whereas the proline content (µg g^−1^ fw) was assessed as described by Toscano et al. [41].

## 3. Results

**Figure 1:**
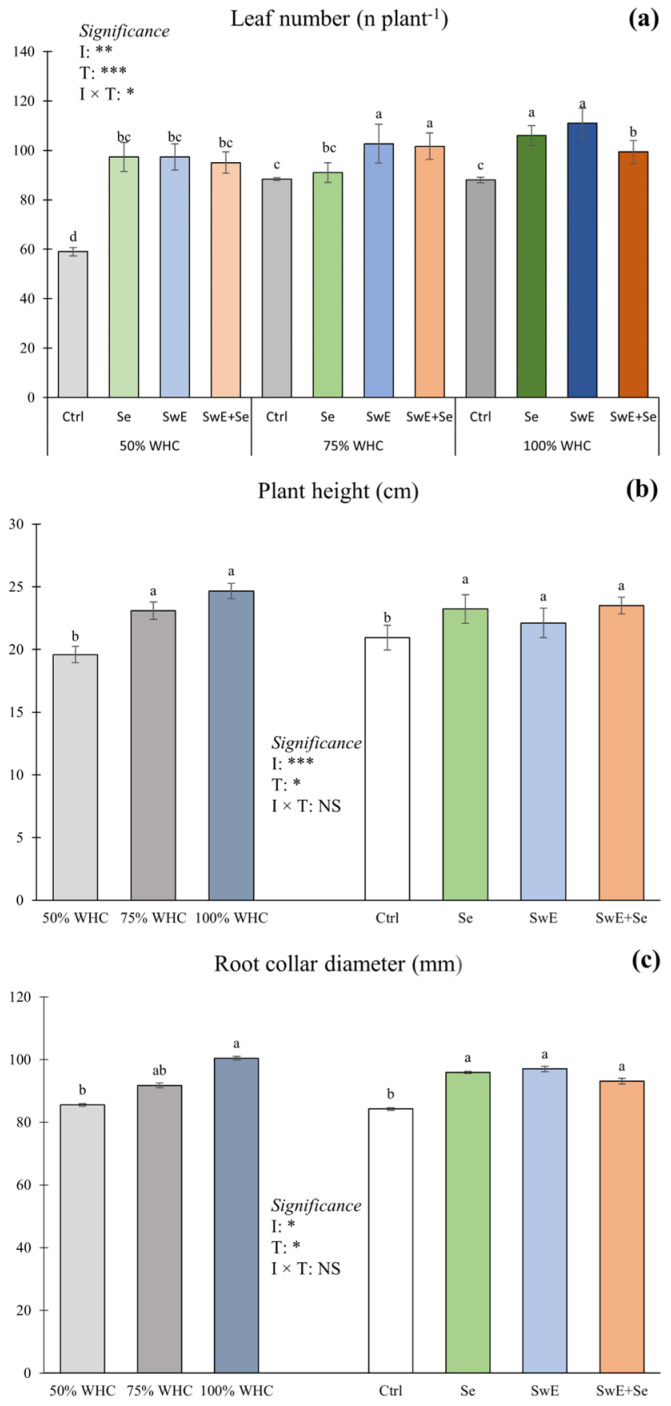
Leaf number (**a**), plant height (**b**) and root collar diameter (**c**) of curly endive affected by irrigation management (50%, 75% and 100% of WHC) and stress alleviators (Ctrl, Se, SwE and Se + SwE). Bars with different letters indicate significant differences (NS: not significant; *: significant at *p* ≤ 0.05; **: significant at *p* ≤ 0.01 ***: significant at *p* ≤ 0.001) and capped vertical lines are standard errors.

For leaf number, ANOVA showed a meaningful effect of the interaction between the irrigation rate and alleviator treatments (Figure 1a).

ANOVA revealed an interaction between irrigation management and drought alleviator treatments for leaf numbers. When compared, both moderate drought stress and well-watered conditions together with SwE and SwE + Se alleviator treatments produced 16.2% and 15.0% more leaves than the control treatments. As expected, the lowest leaf number was recorded in the control treatments subjected to severe drought stress. Nevertheless, plants exposed to severe drought stress but treated with drought alleviators (50% of WHC; Se, SeW, SeW + Se) produced 66.7% more leaves than the control treatment (50% of WHC), revealing a possible overlapping effect between biostimulation/biofortification and water application. As leaf number is one of the main compounds for yield determination, this is a promising result that can help us understand the physiological pathway for yield determination in chicory plants.

Plant height (Figure 1b) and root collar diameter (Figure 1c) were not significantly influenced by the interaction between the two main factors. Averaged over alleviator treatments, plants from 75% and 100% WHC plots displayed a higher plant height compared to those from 50% WHC plots (+17.9 and +25.9% compared to 50% WHC). Regardless of drought stress levels, curly endive from all alleviator treatments showed a higher plant height compared to the control plants (+10.8%, +5.6% and 12.2% compared to the control, respectively) (Figure 1b). For root collar diameter, disregarding treatments, plants exposed to 100% WHC exhibited the highest values (+17.4% compared to 50% WHC), whereas the lowest value was recorded in plants grown under severe drought stress (50% of WHC). Without considering drought stress, alleviator treatments enhanced root collar diameter compared to the control plots. Increases of 13.8%, 15.2% and 10.5% were recorded, respectively, in Se-, SwE- and Se + SwE-treated curly endive compared to non-treated plot plants.

**Figure 2:**
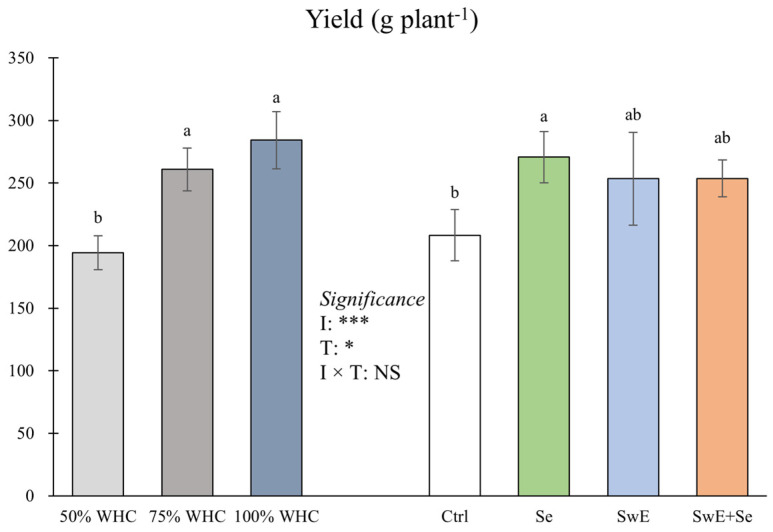
Yield of curly endive affected by irrigation management (50%, 75% and 100% of WHC) and stress alleviators (Ctrl, Se, SwE and Se + SwE). Bars with different letters indicate significant differences (NS: not significant; *: significant at *p* ≤ 0.05; ***: significant at *p* ≤ 0.001) and capped vertical lines are standard errors.

As shown in Figure 2, there was no significant interaction between irrigation management and drought alleviators for yield.

Averaged over the alleviator treatments, curly endive grown under moderate drought stress and optimal water availability conditions showed the highest yield compared to those grown under severe drought stress. Indeed, well-watered plants and plants under moderate drought stress yielded between 34.2 and 46.2% more than plants under severe drought stress.

**Figure 3:**
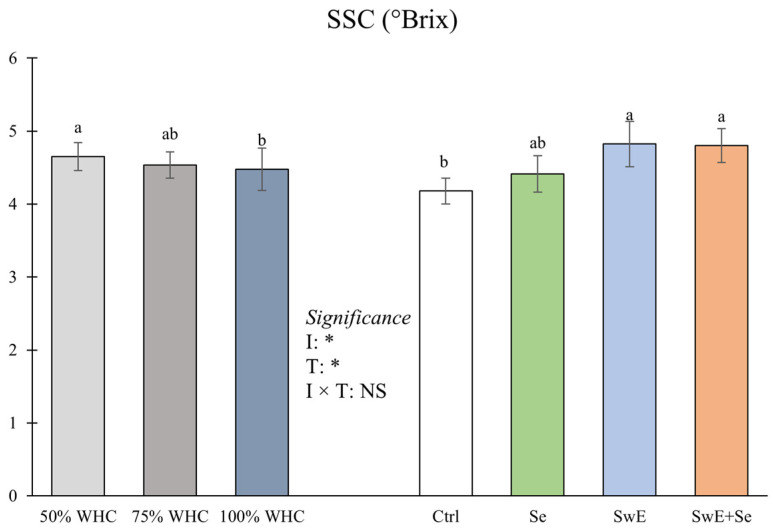
Soluble solid content (SSC) of curly endive affected by irrigation management (50%, 75% and 100% of WHC) and stress alleviators (Ctrl, Se, SwE and Se + SwE). Bars with different letters indicate significant differences (NS: not significant; *: significant at *p* ≤ 0.05;) and capped vertical lines are standard errors.

Considering the alleviator treatments, the highest yield was reported in plants that underwent Se treatments (+30.0% compared to the untreated control), while the lowest was reported in the control plants. Moreover, the yields from Se and SwE + Se plants do not differ statistically from the control or Se treatments. Concerning SSC, ANOVA did not indicate a significant interaction between the two main factors (Figure 3).

Considering the drought stress levels, the highest SSC was found in curly endive exposed to 50% WHC. Conversely, the lowest SSC was recorded in plants subjected to 100% WHC. Averaged over the irrigation levels, plants supplied with SwE or with SwE + Se showed a higher SSC compared to the control plants (+15.4% and +14.9%, respectively). The lowest values were recorded in untreated plants.

As reported in Table 1, the statistics did not show a significant interaction between the two factors for the dry matter content and mineral profile of curly endive.

Regarding dry matter content, the statistics evidenced a significant influence of the drought stress level and stress alleviator treatments.

Averaged over the treatments, the highest values were recorded in plants grown under severe drought stress (50% of WHC), whereas the lowest ones were found in plants subjected to 75% and 100% of WHC. Concerning the alleviator treatments, the highest dry matter content was recorded in untreated plants, while the other treatments displayed the lowest values.

For K content, no significant interaction between irrigation management and drought alleviators was observed, nor was one reported between drought alleviators and K content. In contrast, there was a significant interaction between irrigation management and K content. The lowest K value was found for well-watered management and the highest for severe and moderate drought management (Table 1).

The statistics showed that the main factors did not significantly influence N, P, Ca, Mg and S content in curly endive plants (Table 1).

**Figure 4:**
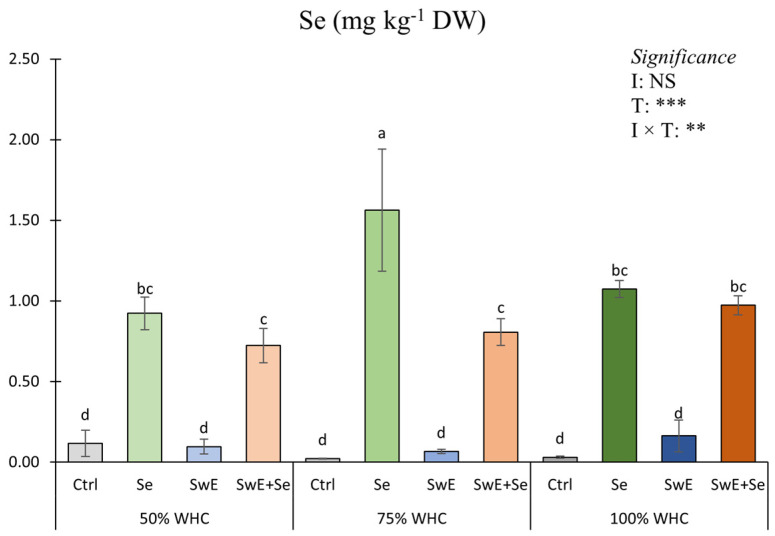
Se concentration of curly endive affected by irrigation management (50%, 75% and 100% of WHC) and stress alleviators (Ctrl, Se, SwE and Se + SwE). Bars with different letters indicate significant differences (NS: not significant; **: significant at *p* ≤ 0.01; ***: significant at *p* ≤ 0.001) and capped vertical lines are standard errors.

Selenium concentration in curly endive tissues was significantly influenced by the interaction between irrigation levels and alleviator treatments (Figure 4).

As shown in Figure 4, Se concentration in plants grown at a medium drought stress level (75% of WHC) and supplied with Se was higher, followed by that detected in plants exposed to the 100% WHC × Se combination. As expected, the lowest values were found in plots not treated with selenium.

**Figure 5:**
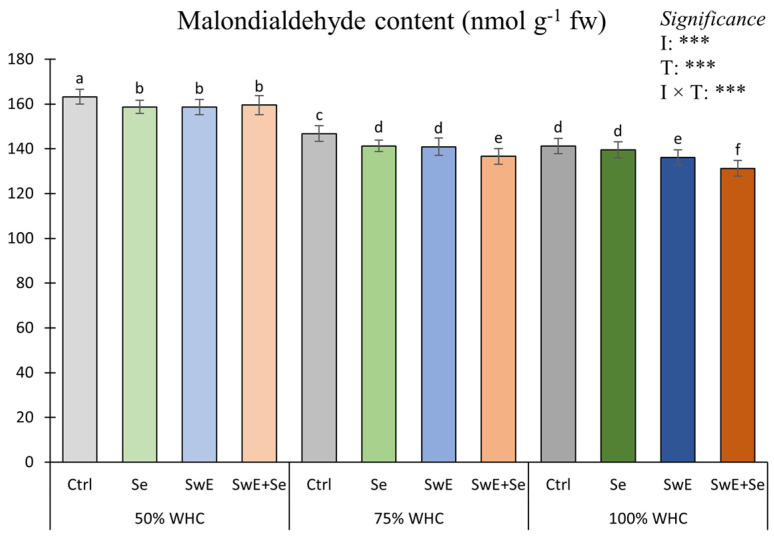
Malondialdehyde concentration of curly endive as affected by drought stress levels (50%, 75% and 100% of WHC) and stress alleviators (Ctrl, Se, SwE and Se + SwE). Ctrl: control; SwE: seaweed extract; Se: selenium. Means with different letters are significantly dissimilar according to Tukey’s HSD test. Bars indicate mean values ± standard error. ***: significant at *p* ≤ 0.001.

Malondialdehyde content was significantly affected by the I × T interaction (Figure 5).

The results indicated that the highest malondialdehyde content was recorded in plants from 50% WHC × Ctrl plots followed by those from the 50% WHC × Se, 50% WHC × SwE and 50% WHC × Se + SwE combinations. The lowest value was recorded in plants from plots cultivated at 100% of WHC and supplied with SwE + Se.

**Figure 6:**
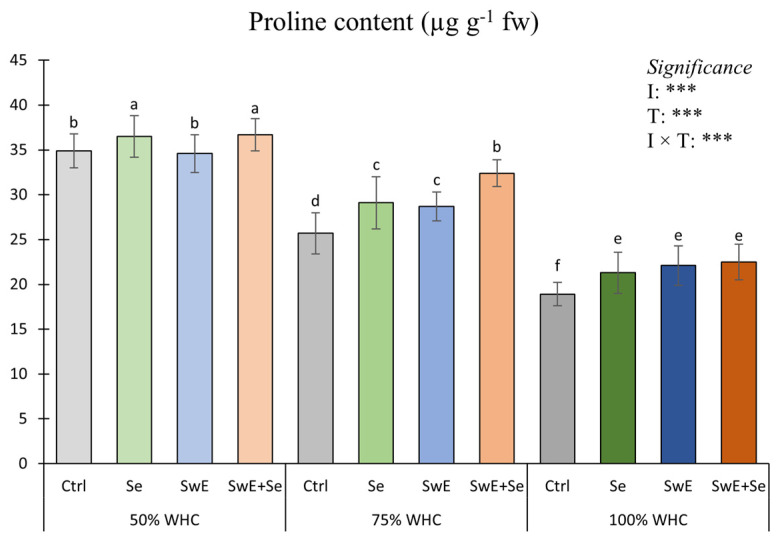
Proline concentration of curly endive as affected by drought stress levels (50%, 75% and 100% of WHC) and stress alleviators (Ctrl, Se, SwE and Se + SwE). Ctrl: control; SwE: seaweed extract; Se: selenium. Means with different letters are significantly dissimilar according to Tukey’s HSD test. Bars indicate mean values ± standard error. ***: significant at *p* ≤ 0.001.

As shown in Figure 6, ANOVA highlighted a significant effect of the I × T interaction.

The highest proline values were recorded in plants from the 50% WHC × Se combination and in those from the 50% WHC × SwE + Se combination followed by plants grown under severe drought stress conditions (50% WHC) not treated and SwE-treated. The lowest proline value was found in control plants grown under optimal water availability conditions (100% WHC).

**Figure 7:**
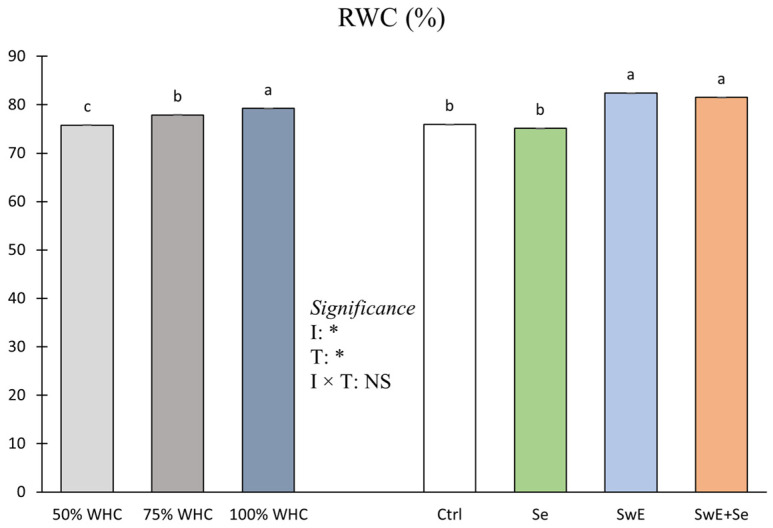
Relative water content (RWC) of curly endive affected by irrigation management (50%, 75% and 100% of WHC) and stress alleviators (Ctrl, Se, SwE and Se + SwE). Bars with different letters indicate significant differences (NS: not significant; *: significant at *p* ≤ 0.05) and capped vertical lines are standard errors.

Considering RWC values, ANOVA did not underline a statistically significant effect of the interaction between the two main factors (Figure 7).

Considering the irrigation levels, the highest RWC values were recorded in plants exposed to 100% of RWC while the lowest ones were found in plots subjected to 50% WHC. On the other hand, SwE or SwE + Se treatments guaranteed higher value in terms of RWC compared to Ctrl or Se-treated plants.

## 4. Discussion

Climate change has become an imperative issue in agriculture since it alters the seasonal cycles, increasing the frequency of unusual long periods of high temperatures and drought, with critical consequences for global food security. For this reason, agriculture research has focused on the development of new eco-friendly tools capable of increasing crop tolerance to abiotic stresses, such as drought. Selenium (Se) is a fundamental micronutrient, which has also been used as a plant drought stress alleviator, thanks to its ability to increase the antioxidant status of treated plants. At the same time, non-microbial biostimulants, such as seaweed extract (SwE), are widely used in the agricultural sector to enhance plant tolerance to drought conditions. In the present study, curly endive plants were cultivated in pots under three different water levels (50%, 75% or 100% of substrate water holding capacity) and were subjected to different drought reliever treatments (Ctrl, SwE, Se or SwE + Se) to understand the implications on plant growth and yield features, nutrient concentrations, relative water content, proline, malondialdehyde, and selenium. Yield, plant height, root collar diameter and leaf number were significantly reduced by drought stress. The data are coherent with those reported by Dietz et al. [43] and Hussain et al. [44]. As documented by the previous scientific literature, the first plant reaction to drought stress is the closure of stomata, reducing CO_2_ assimilation and, consequently, the photosynthesis rate [45]. In our study, an increase in these parameters was recorded when plants were treated with SwE, Se or SwE + Se. The positive effect given by Se application is related to its role in photosynthesis optimization [46]. Moreover, it was reported that Se application can increase cell elongation and nutrient use efficiency [47]. Several studies on leafy vegetables underlined a positive effect of Se biofortification on plant growth and yield traits of lettuce [48,49] and basil [50]. Regarding the effects of SwE, it is widely reported that the application of this biostimulant can encourage productive and growth features of plants [51,52,53]. The principal mechanisms involved concern the effects of natural phytohormones comprised in SWE, which stimulate cell division and elongation, and the activation of plant primary metabolism [53]. Interestingly, in our study, both SwE and Se alone had similar effects on yield, plant height and root collar diameter to the combination SwE + Se, suggesting no synergistic, antagonistic or additive effects between these two treatments. As for leaf number, the results revealed that SwE and Se determined the peak of this parameter in well-watered plants (100% WHC), whereas in moderate water-stressed plants (75% WHC), the peak was recorded in SwE- and in SwE + Se-treated plots. These differences suggest that the single effect of Se treatment is more pronounced only in well-watered plants, whereas in moderate water stress conditions, SwE and the SWE + Se combination showed their efficacy in sustaining plant growth. However, when severe drought stress was imposed (50% WHC), no treatment proved to be better than any other for the leaf number increase, statistically showing the same increase compared to the control plots.

Soluble solid content was enhanced by drought and by SwE, Se or SwE + Se supply. These data overlap with those of Babita et al. [54] and Ahmadi-Mirabad et al. [55]. The increase in soluble solids is often recorded in drought-stressed plants mainly for two reasons: the dehydration of the tissues and the build up of osmo-protective compounds. Water stress, indeed, reduces water uptake, causing a dehydration of the tissues and a greater concentration of soluble solids [56]. Furthermore, water-stressed plants accumulate osmotic solutes, such as sugars, to defend themselves from osmotic stress. This mechanism is called osmotic adaptation, and it helps to maintain cell turgor when drought stress occurs [57,58]. On the other hand, it is reported that Se stimulates antioxidant enzyme activity, reducing oxidative stress and promoting photosynthesis, which in turn enhances sugar biosynthesis in plant [59]. In addition, Se supply boosts the biosynthesis of plant osmo-protective compounds, such as sugars and amino acids [60]. The encouraging effect of SwE on SSC was similarly found by Jalali et al. [61] on tomato plants supplied with SwE. This effect is linked to different mechanisms, such as the stimulation of photosynthesis and, consequently, the accumulation of sugars [62], the increase in nutrient uptake via the better growth of roots [25,61], and the elicitation of plant defense mechanisms against drought stress [63], such as via the biosynthesis of osmo-protective compounds (sugars and amino acids). In our study, the treatments SWE and SwE + Se were statistically similar; consequently, we can assume that no synergistic/antagonistic effects occurred.

Dry matter content peaked in plants grown with a water level of 50% WHC. The data are in accordance with those of Consentino et al. [19]. The increase in dry matter percentage in water-stressed plants is a very common outcome essentially for two reasons: the build up of solutes as a drought consequence [64] and the condensing effect caused by water rest [19]. This study also indicated that SwE, Se and SwE + Se supply significantly reduced dry matter content. Data are coherent with those of Consentino et al. [19], who assume that the increase in water uptake prompted by SwE application explains the decrease in dry matter content. Moreover, the decrease in dry matter found in the plots exposed to the single Se application could be linked to the cell water retention increase [65] and to the rapid enhancement of plant growth [66] stimulated by Se application. As for dry matter content, no interaction between SwE and Se was recorded.

Potassium concentration was enhanced by water stress, but it was not affected by the other treatments. Potassium is a key ion in the osmotic homeostasis of plant cells, since it helps plants to maintain turgor [67]. During water stress conditions, plants accumulate K+ ions to cope with osmotic stress [68]; consequently, the recorded increase can be explained by a plant defense mechanism against water shortage conditions.

Nitrogen content was not influenced by the irrigation regimes and by the drought alleviator treatments. Nitrogen has a key role in plant development, and it is one of the main constituents of several biomolecules. As stated by Xia et al. [69], drought alters nitrogen metabolism, causing a different distribution of nitrogen in plant tissues. Seaweed extract did not affect total nitrogen in curly endive. We hypothesize that biostimulants affected nitrogen metabolism by improving nitrogen enzyme activity, causing a different N balance without, however, affecting the total nitrogen tissue. To be specific, SwE significantly improved growth and, consequently, plant biomass [51]. Considering this result, we might assume that N content percentage can remain unchanged, associating these results with a “dilution” effect. Moreover, Se supply did not affect nitrogen concentration in curly endive. Se is a key micronutrient that modulates plant metabolism and oxidative damage responses [70,71]. Similar results were found by Ferrarese et al. [72] in spinach plants, while, as reported by Puccinelli et al. [73], Se biofortification increases nitrate content in lettuce. These results indicate a species-dependent effect of Se application. Moreover, as reported by Rios et al. [47], Se supply significantly enhances several enzymes involved in nitrogen metabolism (e.g., nitrate reductase, nitrite reductase). Thus, Se could improve N metabolism as well as N utilization, translocation and availability in plants without, however, causing an increase in the total N content.

Our study indicated that P, Ca, Mg and S content in tissues was not influenced by irrigation management or by drought alleviators. These results are coherent with those obtained in other species, suggesting that the nutrient concentration (P, Ca, and Mg) varies according to species and/or cultivar [73,74,75,76,77,78].

In this study, Se concentration was influenced by irrigation management regimes and drought alleviators. The highest Se content was found in moderated drought-stressed plants. These outcomes could be related to an abiotic elicitor, such as moderate drought stress that enhances Se uptake, translocation and accumulation in tissues, considering that Se is involved in antioxidant protection [36]. Similar outcomes were found by Ali et al. [79] and Song et al. [80] on Okra and tomato, where Se treatments increased antioxidant activity in stressed plots. Moreover, it is interesting to report that, under severe drought stress conditions, low transpiration levels could limit Se absorption, reducing its concentration in leaf tissues.

Malondialdehyde (MDA) content is considered as a reliable marker of cell membrane stability under stress conditions [81]. It is one of the final compounds of polyunsaturated fatty acid peroxidation in cells, whose content indicates the extent of the damage triggered by drought stress [82]. In this study, MDA decreased as drought stress intensity decreased. This result is in line with that of Patanè et al. [83] and Zhong et al. [36] on tomato grown under drought stress. Moreover, SwE and Se treatments reduced MDA content in curly endive tissues at all drought stress levels. These results concur with those of Sabatino et al. [78] on chicory plants, where it is hypothesized that SwE-treated plants are more resistant to drought and, consequently, less stressed compared to control plants. Moreover, as described by Lanza et al. [84], plants can biosynthesize selenocysteine, a constituent of glutathione involved in plant responses to abiotic stresses. Indeed, glutathione peroxidase is a key antioxidant enzyme that can be influenced by selenium availability under various abiotic stress conditions. Glutathione peroxidase and glutathione act as scavengers of hydroperoxides. Furthermore, as reported by Zhong et al. [36], Se supply can improve antioxidant enzyme activity, including peroxidase, superoxide dismutase, and catalase, involved in ROS scavenging.

The result evidenced that proline concentration was modified by irrigation management and drought alleviators. In line with several authors [83,85], proline values were higher in plants subjected to severe drought stress compared to those grown under mild drought stress or under optimal water availability. Proline is a common osmolyte that accumulates in plant tissues under a water deficit to reduce cell damage [86]. Accordingly, with Patanè et al. [83] and Desoky et al. [87], proline accumulation could be related to an osmotic adjustment of stressed plants to overcome oxidative damage stimulated by water deficiency. Concomitantly, alleviator treatments determined an increase in proline content. Previous reports indicated that SwE supply increases proline content in plants [85,88,89]; these results could be linked to an elicitor effect and consequently to an increase in antioxidant responses induced by biostimulants and Se application [48,90].

Proline content was affected by irrigation management and drought alleviators. In line with previous studies carried out by Patanè et al. [83] and Goñi et al. [85], we observed that proline content is higher in plants subjected to severe drought management at any level of the drought alleviator when compared with ww and mds. This could be related to an osmotic adjustment of stressed plants to mitigate oxidative damage. This trend was also reported by Desoky [87]. Under severe drought stress, SwE and SwE + Se addition seemed to be the most performant formula, which agrees with previous reports [83,86,87]. This is presumably linked to an elicitor effect that triggers an antioxidant cell response in stressed plants.

Relative water content was affected by drought and treatments. These outcomes are in line with Zahedi et al. [91] on strawberry grown under drought. Since the first plant response to water deficit is stomatal closure to minimize water loss, the RWC alteration can be linked to drought stress plant adaptation [91]. Moreover, in agreement with the research of Xu et al. [23] on spinach, SwE treatments increased the RWC of plants grown under drought stress, indicating that the SwE application might enhance leaf water relations and help preserve cell turgor pressure and expansion. Furthermore, Se supply supported intracellular osmotic equilibrium by the build up of organic osmoregulatory substances and inorganic ion content affecting leaf relative water content [92]. Based on these results, Se treatments represent a useful strategy for supporting osmotic pressure equilibrium as a result of low water availability.

## 5. Conclusions

In the present research, we investigated the single and combined effect of two stress alleviators, such as seaweed extract (SwE) and selenium (Se), on the performance of curly endive cultivated under different irrigation management conditions. Both SwE and Se enhanced leaf number compared to the control under both optimal and suboptimal water availability conditions. The application of SwE + Se enhanced plant height, root collar diameter, yield, SSC and proline compared to the control plots and reduced malondialdehyde content. Based on these results, the mutual application of Se and SwE is a key strategy to overcome moderate drought stress (75% WHC) affecting curly endive plants, ensuring satisfactory productive and qualitative traits. Future studies will evaluate if the recorded effects on the soilless system can be reproduced in large-scale open-field conditions and will deepen the molecular mechanisms behind plant responses.

## Figures and Tables

**Table 1:** Dry matter, K, N, P, Ca, Mg and S content of curly endive as affected by irrigation management (I) (50%, 75% and 100% of WHC) and drought alleviator treatments (T) (Ctrl, Se, SwE and Se + SwE).

Treatments	Dry Matter Content (%)		K (g kg^−1^ dw)		N (g kg^−1^ dw)		P (g kg^−1^ dw)		Ca (g kg^−1^ dw)		Mg (g kg^−1^ dw)		S (g kg^−1^ dw)	
I														
50% WHC	8.67	a	28.11	a	12.56	a	2.95	a	13.74	a	2.51	a	3.39	a
75% WHC	8.06	b	28.49	a	13.37	a	3.18	a	14.29	a	2.68	a	3.45	a
100% WHC	7.94	b	25.27	b	13.04	a	2.95	a	14.63	a	2.78	a	3.62	a
T														
Ctrl	8.51	a	28.16	a	13.30	a	3.08	a	14.66	a	2.61	a	3.18	a
Se	7.93	b	28.80	a	13.51	a	2.95	a	14.62	a	2.76	a	3.84	a
SwE	8.11	b	26.00	a	12.58	a	2.97	a	13.36	a	2.57	a	3.43	a
SwE + Se	8.23	b	26.20	a	12.56	a	3.10	a	14.23	a	2.68	a	3.50	a
Significance														
I	**		*		NS		NS		NS		NS		NS	
T	*		NS		NS		NS		NS		NS		NS	
I × T	NS		NS		NS		NS		NS		NS		NS	

Ctrl: control; SwE: seaweed extract; Se: selenium. Different letters mean significant statistical differences between mean values at 0.05 of probability (Tukey’s HSD test). *: significant at *p* ≤ 0.05; **: significant at *p* ≤ 0.01. NS: not significant.

## Data Availability

The data of this study are available on request.

## Supplementary Materials

The following supporting information can be downloaded at https://www.mdpi.com/article/10.3390/plants15010170/s1, Figure S1: Relative humidity, maximum and minimum temperatures recorded during the cultivation cycle.
