# Supplementary material for: Selenium Biofortification and an Ecklonia maxima-Based Seaweed Extract Jointly Compose Curly Endive Drought Stress Tolerance in a Soilless System
Source: Plants (Basel). 2026 Jan 5;15(1):170. doi: 10.3390/plants15010170 (PMC12787924; doi:10.3390/plants15010170)
Supplement: Supplementary file 1 [file plants-15-00170-s001.zip › plants-4067415-supplementary.pdf]

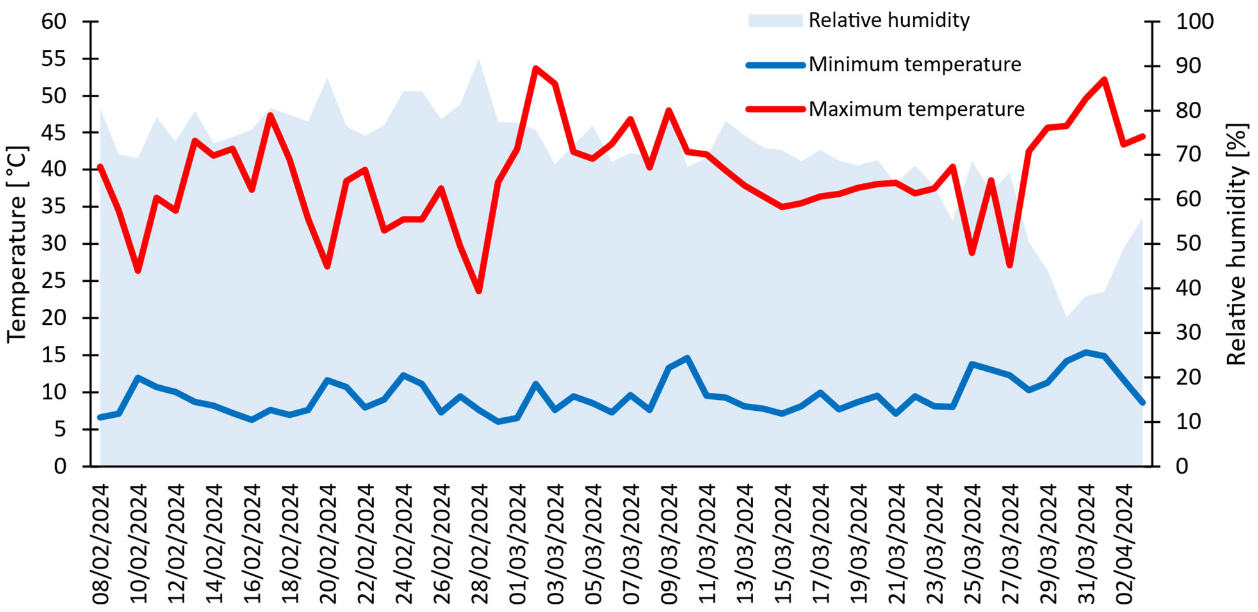

Figure S1. Relative humidity, maximum and minimum temperatures recorded during the cultivation cycle.
